# The Association between Dietary Pattern, Obesity, and Glycemic Control of Children and Adolescents with Type 1 Diabetes Mellitus

**DOI:** 10.3390/nu16030364

**Published:** 2024-01-26

**Authors:** Letícia Victoria Souza da Cunha, Dayan Carvalho Ramos Salles de Oliveira, Leticia de Oliveira Cardoso, Daniela Saes Sartorelli, Beatriz Xavier Peniche, Beatriz Bastos de Araujo, Jorge Luiz Luescher, Raquel Nascimento Chanca Silverio, Alberto Davalos, Patricia de Carvalho Padilha

**Affiliations:** 1 Instituto de Nutrição Josué de Castro (INJC/UFRJ), Rio de Janeiro 21941-590, Brazil; leticiacunhanutri@gmail.com (L.V.S.d.C.); beatrizxpeniche@gmail.com (B.X.P.); beatriz.bastos.a@gmail.com (B.B.d.A.); raquelnascimentocs@gmail.com (R.N.C.S.); 2 Instituto de Puericultura e Pediatria Martagão Gesteira (IPPMG), Universidade Federal do Rio de Janeiro (UFRJ), Rio de Janeiro 21941-590, Brazil; luescher_@hotmail.com; 3 Fundação Oswaldo Cruz (FIOCRUZ), Escola Nacional de Saúde Pública (ENSP), Av. Brasil, 4365-Manguinhos, Rio de Janeiro 21040-360, Brazil; dayanoliv@gmail.com (D.C.R.S.d.O.); leticiadeoliveiracardoso@gmail.com (L.d.O.C.); 4 Departamento de Medicina Social, Faculdade de Medicina de Ribeirão Preto (FMRP), Universidade de São Paulo, Campus da USP—Cidade Universitária, Ribeirão Preto 14040-900, Brazil; daniss@fmrp.usp.br; 5 Divisão de Alimentação e Nutrição (DAN), R. Prof. Marcos Waldemar de Freitas Reis s/n—Campus Gragoatá—São Domingos, Universidade Federal Fluminense (UFF), Niterói 24210-201, Brazil; 6 Instituto Madrilleño de Alimentación (IMDEA), Crta. de, Carr. de Canto Blanco, nº8, E, 28049 Madrid, Spain; alberto.davalos@imdea.org

**Keywords:** diabetes, type 1 diabetes, child, adolescent, dietary pattern, glycemic control, overweight

## Abstract

Aims: To evaluate the association between dietary patterns, obesity, and glycemic control in children and adolescents with type 1 diabetes mellitus (T1DM). Methods: A cross-sectional study was carried out in 2015 at a diabetes reference center in Rio de Janeiro. Sociodemographic data and those related to outpatient follow-ups were obtained from the medical records. The assessment of food consumption was performed using a 24 h food recall. Obesity was defined as body mass index-for-age (BMI-for-age) ≥ +1 z-score. Glycemic control was assessed using glycated hemoglobin (HbA1c). Dietary patterns were generated by factorial analysis, and each individual’s adherence to these dietary patterns was determined by the factor loadings and then classified into terciles. Results: The study population included 120 children and adolescents, among whom 5 dietary patterns were identified. The prevalence of obesity was 31.7% (*n* = 38), and 64.2% (*n* = 77) of the participants had inadequate glycemic control. We observed that individuals with higher adherence to dietary pattern five, characterized by a greater consumption of ultra-processed foods, had higher odds of having higher HbA1c levels (OR = 3.49; 95% CI = 1.18–11.16). Conclusions: Higher consumption of ultra-processed foods can be detrimental to glycemic control in children and adolescents. Thus, food intake monitoring is of paramount importance as part of the multidisciplinary care of patients with T1DM.

## 1. Introduction

Diabetes mellitus (DM) is a metabolic disorder characterized by persistent hyperglycemia, caused by a deficiency in insulin production, action, or both mechanisms. The American Diabetes Association (ADA) classifies diabetes mellitus into type 1 (T1DM), type 2 (T2DM), other specific types of diabetes mellitus, and gestational diabetes mellitus (DMG) [1]. T1DM is the most common endocrinopathy in children and adolescents [2], and is caused by the complete deficiency in insulin production due to the destruction of pancreatic β cells through an autoimmune disease response [1,3]. Incidences of T1DM are increasing [2], and it requires continuous clinical follow-up by a multidisciplinary team to both prevent acute and chronic complications and improve patients’ quality of life [1,4].

The prevalence of obesity among children and adolescents is increasing globally, primarily due to a sedentary lifestyle and changes in dietary patterns [5,6,7]. Multiple studies worldwide have reported an obesity prevalence ranging from 32 to 36% in their population [6]. Research suggests that children and adolescents with T1DM follow similar unhealthy dietary practices of children in the general population, which may lead to obesity [5,8,9]. Children’s dietary profiles often exhibit high energy density through the replacement of fiber-rich foods for highly processed foods, which are rich in fats and sugars [5,8]. Children with T1DM may have a similar dietary profile, consume processed foods that are poor in fiber and have low nutritional value, which results in adverse effects during treatment [9]. Ultra-processed foods (UPA), defined as industrial formulations manufactured from substances derived from foods and additives with minimal amounts of whole foods, have a high glycemic load, which may increase the risk of metabolic syndrome in genetically predisposed children, accelerating the destruction of beta-cells, which are responsible for insulin production, and consequently, causing an early onset of T1DM [9,10,11]. Additionally, unhealthy dietary practices are often associated with the development of non-communicable diseases (NCDs), higher risk of mortality, and metabolic disorders [8,12].

Studies demonstrated that the consumption of refined grain products, desserts, chips, and sugary drinks together represented almost half of the total daily energy intake. The results found show that better diet quality is associated with lower BMI percentiles, but no statistically significant association was found between diet quality and glycemic control assessed by HbA1c [8,12,13].

The risk of cardiovascular disease (CVD) is increased in patients with diabetes. The main risk factors are: duration of diabetes, obesity or being overweight, hypertension, dyslipidemia, smoking, family history of premature coronary heart disease, chronic kidney disease (CKD), and presence of albuminuria. The management of glycemia, blood pressure, and lipids, as well as the introduction of specific therapies for cardiovascular and renal outcomes, are considered essential elements in reducing overall risk. Studies using different methodologies show that young people with type 1 diabetes can have subclinical CVD within the first decade of diagnosis [1].

Given this context, it is important to investigate the relationship between dietary practices and T1DM outcomes, especially those related to micro and macrovascular complications of the disease, such as glycemic control and nutritional status. Dietary pattern derivation is a practical method to evaluate dietary practices along with the complex relationships between diverse foods consumed, unlike isolated analyses of foods and nutrients [13]. Therefore, the objective of this study was to evaluate the association between dietary patterns, obesity, and glycemic control in children and adolescents with T1DM treated at a university hospital in Rio de Janeiro.

## 2. Methods

### 2.1. Study Sample, Locationof the Study, and Eligibility Criteria

This is a cross-sectional study in which the database originated from the project “diet quality and its association with the nutritional status and metabolic control of children and adolescents with type 1 diabetes mellitus”, and was conducted by STROBE checklist recommendation. This database consisted of 120 children and adolescents treated at a referral service in Rio de Janeiro, Brazil. Instituto de Puericultura e Pediatria Martagão Gesteira (IPPMG/UFRJ) is characterized by being a research, teaching, and healthcare institution, created to promote the health and quality of life of children and adolescents, in addition to contributing to the qualification of professionals in the area. This place is also one of the major referral centers in Rio de Janeiro.

The IPPMG/UFRJ Diabetes Outpatient Clinic serves approximately 200 patients monthly and is a reference center for the treatment of DM1 in children and adolescents in the city of Rio de Janeiro. The outpatient team is characterized by multi-professionality and consultations are integrated and carried out by doctors, nutritionists, psychologists, and nurses.

Eligibility criteria were: (1) age between 7 and 16 years; (2) diagnosed with DM1 in the past year; (3) absence of another autoimmune disease, such as celiac disease; and (4) information on study results (glycemic control and nutritional status). Children/adolescents who refused to do the 24 h food recall were excluded.

### 2.2. Sample Size

The definition of the sample size was based on the original study and aimed to detect a difference of 1.1% in glycated hemoglobin, considering a standard deviation of 2.0%, alpha error of 5%, and test power of 80%, estimating that the sample size should be at least 41 children.

### 2.3. Outcomes (Dependent Variables)

#### 2.3.1. Anthropometric Measurements

BMI was calculated by dividing the weight, in kilograms, by the square of the height, in meters. The body weight was measured by a PL 180 digital scale (150 kg of maximum capacity and 0.1 kg of accuracy), manufactured by FILIZOLA^®^, São Paulo, Brazil. The measurement was taken with the child or adolescent standing upright, barefoot, looking straight ahead, remaining relaxed, and wearing lightweight clothing. Height was obtained (in meters) using a TONELLI^®^ stadiometer with a precision of 0.1 cm. The individuals were positioned standing on a scale platform, barefoot, with the head in the Frankfurt plane, knees not flexed, heels, buttocks, and scapulae in contact with the vertical surface of the stadiometer, with the palms of the hands in contact with the hips. Height was measured in duplicate, to reduce intra- and interpersonal variations, and the average was calculated [14].

The BMI-for-age was classified according to the cutoff points proposed by the World Health Organization (WHO): (1) thin (<−2 z-score); (2) eutrophic (≥−2 and <+1 z-score; (3) overweight (≥+1 and <+2 z-score); and (4) obesity (>+2 z-score) [14]. For the calculations, WHO AnthroPlus software version 3.2 was used [15,16]. The assessment of BMI was dichotomized into not overweight (<+1 z-score BMI/age) and overweight (≥+1 z-score BMI/age).

#### 2.3.2. Glycemic Control

Glycemic control was evaluated according to the value of glycated hemoglobin (HbA1c). The variable was evaluated categorically using the cutoff points proposed by the Brazilian Society of Diabetes and the American Diabetes Association, considering HbA1c < 7.5% as a desirable value for children and adolescents with DM1 [1,17]. To determine the percentage of glycated hemoglobin, (HbA1c%) the Endpoint test, VITROS 5600, 4600, 5.1, FS system was used.

To prevent complications related to diabetes, HbA1c values below 7% are recommended for individuals with diabetes, and this target can be individualized and readjusted throughout treatment. However, for children and adolescents, some patients are recommended to have less rigid glycemic targets (7.5%) for reasons such as: not being able to deal with symptoms of hypoglycemia or presenting asymptomatic hypoglycemia; being given limited access to diabetes technologies and analog insulins and/or continuous glucose monitoring; and not being able to check blood glucose regularly or having non-glycemic factors that increase HbA1c [1]. It was the reason this study considered good control glycemic targets (<7.5%).

### 2.4. Independent Variable

#### 2.4.1. Food Consumption

Food consumption was analyzed by 24 h food recall (R24h). The information collected included portion size, cooking method, time of consumption, food product brand, and specifications (conventional, diet, light, “zero”). The parents/adolescents were instructed to ask about the use of industrialized spices in the preparation of meals. Aphoto album was used to illustrate the size of food portions and utensils to facilitate the correct description of the quantities consumed by the interviewee. TheR24h was used following the five-step multiple-pass method, which is used to encourage participants to remember the foods consumed through five steps: (1) uninterrupted listing of foods and drinks consumed, (2) questioning about foods that are often omitted, (3) time and occasion on which the food was consumed, (4) detailed description of the quantities and other characteristics of the mentioned foods, and (5) to review of the information provided for certification of that there was no omission of any food [18]. Finally, the reported food portions were converted into units of mass and volume using the food consumption assessment table in household measurements [19] and centesimal and energy quantities using the Brazilian table of food composition [20].

#### 2.4.2. Food Grouping Methodology

The analysis of eating patterns considers the relationships between the most diverse foods consumed, which represents the complexity of the combinations, unlike isolated analyses of foods and nutrients [13]. The food items reported by the patients were classified into 13 groups according to similarities in nutritional composition and their possible influence on blood glucose, and are described in Table 1.

### 2.5. Covariates

#### Sociodemographic and Outpatient Follow-Up Data

Sociodemographic data was collected by specific medical record forms. Missing information was collected directly from the participant before the medical appointment. These variables were: child’s age (years), family income (Brazilianreals), place of residence, housing sanitation conditions (adequate or not adequate), parent’s level of education (more or less than 8 years), and family composition (number of residents in the household).

The outpatient follow-up variables were: the child’s age (years), time since diabetes diagnosis (years), and dietary method used (traditional or carbohydrate counting method). The traditional methods were defined according to portions of the equivalents of the food groups, considering the caloric value. All variables related to outpatient follow-up are information collected routinely at the outpatient clinic and not exclusively for the research.

### 2.6. Statistical Analysis

The data were entered and statistical analysis was carried out using the program Stata statistical and data analysis software version 13.1. The significance level adopted was 5%. Categorical variables were expressed by absolute frequency (n) and percentage (%). The normality of continuous variables was checked using the Kolmogorov–Smirnov test, with the parametric expressed through means and standard deviation (SD).

Dietary patterns were derived from factor analysis and extracted by principal component analysis [21,22]. Eigen values greater than one were considered to determine the number of dietary patterns in our analysis. Foods corresponding to each dietary pattern were identified based on their factor loadings, with a cutoff point greater than or equal to 0.2773501 (i.e., the square root of 1 divided by the 13 food groups evaluated). Finally, individual factor scores (i.e., estimates of each individual’s location on the factors) were extracted from the factor analysis. These factors have a z-score distribution (i.e., mean zero, SDone), where higher values signify greater adherence to a given dietary pattern. The distribution of individual adherence to each dietary pattern was recategorized into terciles, which are interpreted as low, medium, and high adherence to a given dietary pattern.

The comparison of these terciles with the different variables analyzed in this study allows us to verify the degree of association between these outcomes and adherence to the different dietary patterns identified [23]. Binomial statistical models were used to estimate the associations between dichotomous outcomes (i.e., excessive weight and glycemic control) and dietary patterns. Models were adjusted for gender, total energy intake, time of diagnosis, insulin dose, and age. Thus, our results show the effect of adherence to dietary patterns regardless of the effects of age, sex, time of diagnosis, insulin dose, and total energy intake [23].

### 2.7. Ethical Issues

All procedures involving study participants were submitted to and approved by the Instituto de Puericultura e Pediatria Martagão Gesteira (IPPMG) Research Ethics Committee at the Federal University of Rio de Janeiro (UFRJ), number: 1.478.806. Written informed consent was obtained from all participants from their legal guardians.

## 3. Results

The present study was comprised of 120 children and adolescents diagnosed with T1DM, who had an average age of 11.7 years (±2.8) and a predominance of females (53.3%, *n* = 64). The average BMI was 19.7 kg/m^2^ and z-score mean of BMI was 0.65 (±0.89), with 31.7% of the sample (*n* = 38) with overweight (overweight + obesity) and only 1.7% (*n* = 2) with thinness. As for glycemic control, the average value of glycated hemoglobin (HbA1c) was 8.13% (±1.3), highlighting that 64.2% (*n* = 77) had inadequate glycemic control. In this casuistic, no participant used an insulin pump, only injections of multiple doses per day. Table 2 presents the characterization of the studied population.

The average amount of calories of participants was 1756.4 ± 518.4 kcal, and five dietary patterns were identified from principal component analysis. Table 3 represents the factor loadings obtained in each dietary pattern for all food groups included. The first dietary pattern (DP1) showed a positive factorial load for rice, beans, bread, fruits, vegetables, and greens, indicating a higher consumption of those food groups, and a negative factorial load for tubers, pasta, and flours, indicating a lower consumption of those. The second pattern (DP2) was characterized by the consumption of sugary drinks, meat oils, and fats. The third dietary pattern (DP3) showed a positive factorial load for sausages, oils, and fats, and a negative factorial load for meat. The fourth pattern (DP4) was characterized by a positive factorial load of oils and fats and a negative for sugary drinks. The fifth dietary pattern (DP5) showed a positive factorial load for the snacks group.

Table 4 represents the models adjusted for the association of dietary patterns with overweight and glycemic control. As for nutritional status, in this population, no statistically significant relationship was observed with any of the dietary patterns found.

Concerning glycemic control, we observed that individuals with a higher adherence to DP5, characterized by greater consumption of ultra-processed foods, had higher odds of having high HbA1c levels (OR = 3.49; 95% CI = 1.18–11.16) compared to those with low adherence.

## 4. Discussion

The findings in this study revealed a high prevalence of overweight participants and a significant association between DP5and glycemic control, suggesting that greater consumption of ultra-processed foods was associated with worse outcomes. These suggest an aggravation in the cardiovascular risk factors among children and adolescents since obesity and poor glycemic control represent important markers of cardiovascular risk for individuals with DM1.

The increasing prevalence of obesity is one of the most alarming current health problems, and studies have shown that there is also an increase in obesity among children and adolescents with DM1. The American study SEARCH for Diabetes in Youth found that 34.7% of participants with DM1 were overweight or obese [6], corroborating with Gomes et al. [24] who in a Brazilian multicenter study obtained a prevalence of 25.9% for children who were overweight and 4.4% obese. A Turkish study found a prevalence of overweight children of 28%, with 11.2% being obese in a sample of 110 adolescents with DM1 [25]. These results are in agreement with the information found in the present study (overweight in 31.7% of the sample).

Although no significant associations were found between dietary patterns and excess weight, the prevalence of obesity in the population is still considered high and may be related to increased calorie intake, decreased physical activity [26], and high insulin doses in an obesogenic environment [27]. This information reinforces the need for monitoring DM1 patients by a multidisciplinary team and the need to educate patients and their guardians about the importance of blood glucose monitoring, insulin therapy, physical activity, healthy eating practices, and following nutritional guidelines appropriate for each case individually made by a professional in the area.

There are few studies about type 1 diabetes and the consumption of ultra-processed food (UPF) in the childhood context. The inflammation is associated with many chronic diseases. The global growth in the prevalence of non-communicable diseases in recent years has been accompanied by an increase in the consumption of UPF, which has already been recognized as a risk factor for several chronic diseases and cardiovascular diseases. In people with diabetes, especially children, it is important to know the contribution of these foods to promote low-grade inflammation and thus favor other complications [27,28].

Regarding glycemic control and dietary patterns observed in the sample, a positive association was observed between HbA1c levels and higher consumption of ultra-processed foods. As defined by NOVA, ultra-processed foods are industrial formulations of substances extracted or derived from foods. They contain little or no whole food in their composition and are made up of added flavorings, colorings, emulsifiers, and other additives [29]. Previous studies have observed that ultra-processed foods have a higher energy density, more sugar and fat, and less fiber and protein when compared with non-ultra-processed foods [30,31], in addition to being related to low satiety and inducing high glycemic responses [32].

A study by Liu et al. [33] suggests that as there is an increase in the contribution of ultra-processed foods in total calories consumed, there is a progressive decrease in the consumption of healthy foods and an increase in the consumption of unhealthy foods among children, adolescents, and adults. Additionally, lower family income and lower educational level of those responsible seem to determine the preference for ultra-processed foods over non-ultra-processed foods among Brazilian children and adolescents with DM1 [11]. The increased consumption of ultra-processed foods has been noted among children and adolescents. In Brazil, Louzada et al. [30] found that 20.4% of total energy intake was from ultra-processed foods among children older than 10 years. Regarding DM1, Fortins et al. [34] found that 24.25% of total energy consumed was from ultra-processed foods in a Brazilian study with 120 children and adolescents with DM1.

Şahin Bodur et al. [25] found a trend of increased HbA1c in male patients with DM1 in a study with 110 adolescents, where 5% of the total sample (84 individuals) needed improvements in diet quality. Fritz et al. [35] observed higher consumption of carbohydrates and fats and lower consumption of fiber in patients with DM1 and higher HbA1c levels in 34 children and adolescents with DM1 in a Brazilian study. However, unlike the present study, previous studies found no statistically significant association between increased consumption of ultra-processed foods/quality of diet and higher HbA1c levels [8,35,36]. However, Rocha et al. [11] identified excessive consumption of these foods in the population with DM1, and the determining factors for the choice were lower family income and lower educational level of those responsible.

In the context of food choices, it is also important to highlight the management of carbohydrate counting, recognized as a successful nutritional guidance strategy for glycemic control, but often disseminated without concern for the quality of the diet and its impact on other related clinical diabetes outcomes, such as being overweight. At the center where the research was carried out, carbohydrate counting is guided by qualified nutritionists with a focus on healthy eating and building good eating habits, related to eventual flexibility and based on diabetes education, as suggested by the American Diabetes Association [1].

The sample size may be considered a limitation of the present study, but we tried to deal with this partially by using the individual contribution of each individual within each dietary pattern. The lack of data on physical activity is also a limitation, considering the role of a sedentary lifestyle in increasing rates of excess weight in the pediatric population. This casuistic also included only the application of one R24h to analyze the food consumption. However, the interviewers used the multiple-pass method, which aims to encourage the participants to remember the foods consumed through steps, as described in the methods. In addition, 18.3% (*n* = 22) of the participants had a second R24h to correct the variation in habitual and occasional food intake.

Thus, the total n of the sample was used for the analysis of each dietary pattern. In contrast, the scarcity of studies with similar results, demonstrating the importance of diet quality in glycemic control of children and adolescents with DM1, is a strength of the study and suggests a need for further research on the subject, using data that considers more recent consumption trends and larger sample populations.

## 5. Conclusions

Higher consumption of ultra-processed foods can be detrimental to glycemic control in children and adolescents. Thus, food intake monitoring is of paramount importance as part of the multidisciplinary care of patients with T1DM.

This study endorses the findings in the literature on the importance of monitoring the nutritional status of patients with T1DM and highlights the importance of evaluating dietary patterns in glycemic control. A multidisciplinary approach that includes nutritional guidance is extremely important in the treatment of children and adolescents with T1DM. In this context, the educational nutrition process and raising family awareness about the dietary patterns of children and adolescents with DM1 is essential. There is an urgent need for future work on this topic using current food consumption data and larger shipment samples to endorse or refute the conclusions presented.

The nutritionist is indispensable in providing optimal care and assistance to these patients, considering the importance of nutrition in diabetes assistance, such as maintaining healthy blood glucose levels, maintaining weight, providing adequate energy intake, ensuring adequate growth and development, and preventing diabetes complications.

## Figures and Tables

**Table 1:** Groups and food items reported by study population.

Group	Food Items
Rice	White rice and brown rice
Beans	Black beans with sausages, black beans, and pinto beans
Zero drinks	Sugar-free chocolate powder, sugar-free concentrated grape juice, sugar-free coffee, diet powdered refreshment, sugar-free cola soda, sugar-free natural passion fruit juice, and sugar-free orange juice
Sugary drinks	Ultra-processed milk beverages, chocolate milk, powdered soft drinks, natural fruit juice with added sugar, Sustagen^®^ supplement, and concentrated cashew, orange, and passion fruit juices with sugar
Meat	Beef, beef-based preparations, poultry-based preparations, poultry, fish, and fish-based preparations
Sausage	Sausage, turkey breast, mortadella, nuggets, pâtés, and sausage
Pasta and flour	Angu, farofa, pasta, porridge, oats, and tapioca
Fruits, vegetables, and greens	Zucchini, watercress, plum, eggplant, onion, chicory, cauliflower, guava, papaya, mango, tangerine, corn, pear, okra, cabbage, tomato, grape, squash, lettuce, banana, beetroot, broccoli, carrot, chayote, coconut, cabbage, spinach, kiwi, orange, apple, watermelon, melon, cucumber, and preparations based on fruits or vegetables
Snacks	Candies, potato chips, sweet and savory biscuits, cakes, brownies, cereal bars, chocolates, snacks, milkshakes, pastels, *paçoca*, popcorn, pizza, ice cream, and hot dogs
Dairy products	Natural yogurt, cow’s milk, cheeses, and Greek yogurt.
Oil and fat	Mayonnaise, butter, and margarine
Bread	French bread, pita bread, milk bread, traditional bread, wholemeal bread, refined loaf of bread, cornbread, and toast
Tubers	Yam, french fries, and preparations based on tubers

**Table 2:** Characteristics of children and adolescents with type 1 diabetes mellitus treated at a reference service in Rio de Janeiro (*n* = 120).

Continuous Variables	Average (SD)
HbA1c (%) (*n* = 120)	8.13 (1.26)
Age (years) (*n* = 120)	11.7 (2.88)
Diabetes duration (years) (*n* = 119)	6.68 (3.33)
Child’s age at diagnosis (years) (*n* = 120)	5.07 (2.71)
Insulin dose (units/kg/day) (*n* = 119)	1.05 (0.46)
BMI (kg/m^2^) (*n* = 120)	19.7 (3.71)
Z-score BMI (*n* = 120)	0.65 (±0.89)
Categorical variables	*n* (%)
Sex	
Female	64 (53.3)
Male	56 (46.7)
Places of residence	
Rio de Janeiro	112 (93.3)
Other regions and states	8 (6.7)
Family structure	
Lives with one parent	35 (29.2)
Lives with parents	85 (70.8)
Lives with another guardian	2 (1.7)
Parents’ schooling	
Fundamental	17 (14.4)
High school	77 (65.3)
Higher education	24 (20.3)
BMI	
Thin	2 (1.7)
Eutrophic	80 (66.7)
Overweight	27 (22.5)
Obesity	11 (9.1)
Glycemic control	
Adequate	43 (35.8)
Inadequate	77 (64.2)
Dietary planning methods	
Portion method	24 (20.0)
Carbohydrate counting method	96 (80.0)

SD: standard deviation; BMI: body mass index; HbA1c: glycated hemoglobin.

**Table 3:** Factor loadings obtained by factor analysis in food consumption data of children and adolescents treated at the diabetes mellitus clinic at IPPMG/UFRJ.

Food Groups	DP1	DP2	DP3	DP4	DP5
Rice	0.622 *	0.222	−0.078	0.028	0.12
Bean	0.64 *	0.045	0.031	0.034	0.253
FVG	0.332 *	0.138	−0.086	0.027	−0.229
Meat	−0.002	0.48 *	−0.866 *	0.126	0
Zero drinks	−0.059	−0.068	0.056	0.113	0.21
Sugary drinks	−0.001	0.693 *	0.211	−0.686 *	0
Processed meat	0.039	−0.231	0.319 *	0.015	0.093
Pasta and flour	−0.455 *	−0.094	0.233	0.087	−0.142
Snacks	−0.263	−0.147	−0.034	−0.027	0.655 *
Dairy products	0.166	−0.095	−0.012	0.216	−0.082
Oil and fat	−0.002	0.664 *	0.406 *	0.625 *	0.001
Bread	0.281 *	0.215	0.203	0.25	−0.262
Tubers	−0.378 *	0.003	0.083	−0.067	−0.097

* Foods corresponding to each dietary pattern (identified based on their factor loadings, with a cutoff point greater than or equal to 0.2773501).

**Table 4:** Models of estimates of learning adjusted for gender. Total energy intake reported in the 24 h recall. Time of diagnosis. Insulin dose and age.

Variable	DP1		DP2		DP3		DP4		DP5	
	Tercile 2 *	Tercile 3 *	Tercile 2 *	Tercile 3 *	Tercile 2 *	Tercile 3 *	Tercile 2 *	Tercile 3 *	Tercile 2 *	Tercile 3 *
Nutritional status (BMI/Age)	0.88 (0.33–2.30)	0.55 (0.19–1.56)	0.71 (0.26–1.92)	0.61 (0.22–1.65)	0.73 (0.27–1.94)	0.82 (0.31–2.13)	0.53 (0.19–1.44)	0.83 (0.30–2.28)	0.74 (0.28–1.94)	0.57 (0.20–1.59)
Glycemic control (HbA1c)	1.26 (0.47–3.46)	1.43 (0.52–4.06)	0.76 (0.27–2.11)	0.57 (0.19–1.62)	1.31 (0.46–3.80)	0.51 (0.19–1.34)	0.51 (0.17–1.46)	0.43 (0.14–1.28)	1.47 (0.54–4.08)	3.49 (1.18–11.16) **

* = Confidential interval (CI) 95%. ** = *p* < 0.05. DP: dietary pattern.

## Data Availability

The data presented in this study are available upon request from the corresponding author. The data are not publicly available due to ethical issues.
